# Water Jet–Assisted Lipoaspiration as a Novel Therapeutic Approach for Chronic Morel-Lavallée Lesion: A Case Report

**DOI:** 10.1093/asjof/ojaf132

**Published:** 2025-10-17

**Authors:** Pantea Pour Farid, Elisabeth Eschenbacher, Raymund E Horch

## Abstract

Morel-Lavallée lesions (MLLs) are rare closed soft tissue degloving injuries caused by shear forces that separate subcutaneous tissue from the underlying fascia. Chronic MLLs can lead to persistent functional impairment and aesthetic deformities. Standardized treatment approaches are limited, particularly for long-standing lesions without overlying skin necrosis. We report the case of a 42-year-old female patient with a chronic MLL on the left thigh, >2 years after a traffic accident. She experienced discomfort, abnormal soft tissue mobility, and noticeable thigh asymmetry. MRI revealed a persistent subcutaneous cleft without hematoma or seroma. A water jet–assisted lipoaspiration (WAL) procedure was performed to create multiple subcutaneous tunnels, promoting scar formation and tissue adhesion to stabilize the mobile tissue. The postoperative course was uneventful, and the patient reported significant improvement in both function and appearance. Follow-up MRI demonstrated complete obliteration of the subcutaneous cavity. A second WAL procedure was performed because of residual asymmetry, again with favorable results. This case highlights the potential of WAL to induce scar formation and eliminate pathological dead space, restoring soft tissue stability. The patient experienced complete symptom resolution and improved contour symmetry. WAL may serve as a promising minimally invasive treatment for chronic MLLs. It allows functional restoration and satisfactory cosmetic outcomes while avoiding extensive surgical revision.

**Level of Evidence:** 4 (Therapeutic) 
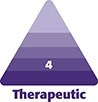

First described in 1853 by Victor–Auguste–François Morel-Lavallée, a Morel-Lavallée lesion (MLL) is a closed traumatic internal degloving injury of the soft tissue caused by significant shear forces, most commonly associated with rollover trauma.^1^

This mechanism leads to a characteristic separation of the dermis and/or subcutaneous fat from the underlying fascia. Awareness of this rare complication is crucial, as diagnosis requires a thorough understanding of the trauma mechanism—an especially challenging task in polytraumatized patients, particularly in the presence of fractures.

According to the literature, >30% of MLLs are initially missed during early examinations.^2^ Typical clinical findings, beyond bruising, include skin hypermobility and abnormal, fluctuating fluid collections accompanied by swelling. Although radiographs and computed tomography scans may aid in identifying associated injuries, ultrasound remains a key tool for detecting MLLs.

Although conservative management is often the first-line approach in the acute phase, interventions such as fluid or hematoma aspiration, debridement, or operative irrigation may become necessary. Chronic lesions and scarring frequently result in cosmetic deformities and functional impairments.

Although liposuction has occasionally been reported as a cause of inadvertent MLL formation, we report here, for the first time, the successful therapeutic use of water jet–assisted lipoaspiration (WAL). In this case, multiple tunnels were intentionally created to induce scarring channels, thereby stabilizing the mobile subcutaneous tissue in a patient with a long-standing MLL.

## CASE REPORT

**Figure 1.**
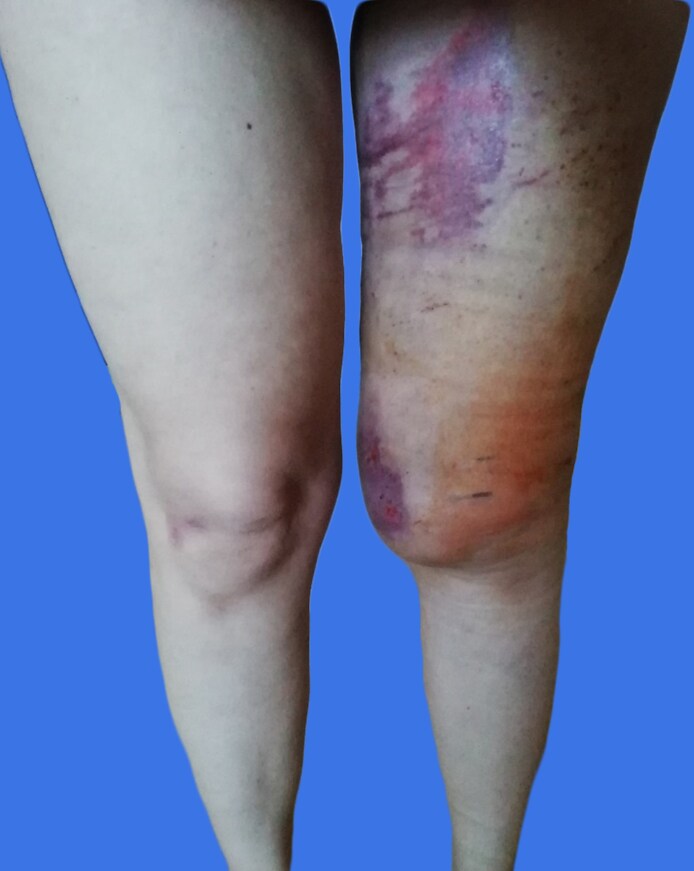
Patient-provided images of the lower extremities of a 42-year-old female after being struck by a motor vehicle as a pedestrian. The background has been edited for privacy/clarity.

A 42-year-old female patient presented to our institution ∼2 years after sustaining a degloving injury, which occurred when she was struck by a car and compressed against a wall in July 2020. According to the patient's history, the accident resulted in skin erosions and laceration wounds on the medial thigh, as well as extensive hematoma formation on the lateral aspect of the left leg, extending from the hip to the knee (Figure 1). Following the rollover trauma, a subcutaneous hematoma evacuation was performed on the left thigh by the trauma surgery department in-house. The fascia of the thigh was found to be intact. Subsequently, 2 drains were placed.

**Figure 2.**
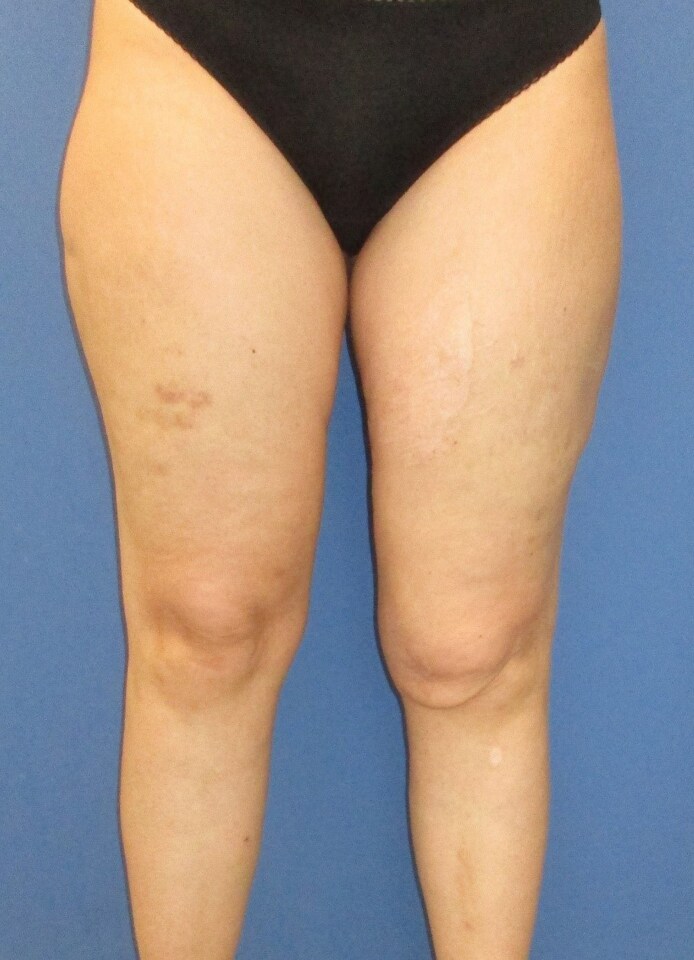
Clinical photograph of a 42-year-old female at the time of first presentation, ∼2 years postinjury. The asymmetry of the thighs, with a noticeable bulging in the proximal lateral region of the left thigh.

At the time of her initial consultation, the patient reported significant functional impairment, including discomfort when walking, running, or sitting, attributed to the abnormal mobility of the skin and subcutaneous tissue. Clinical examination revealed marked asymmetry of the thighs, with a noticeable bulging in the proximal lateral region of the left thigh (Figure 2). Previous attempts at compression therapy had been unsuccessful.

**Figure 3.**
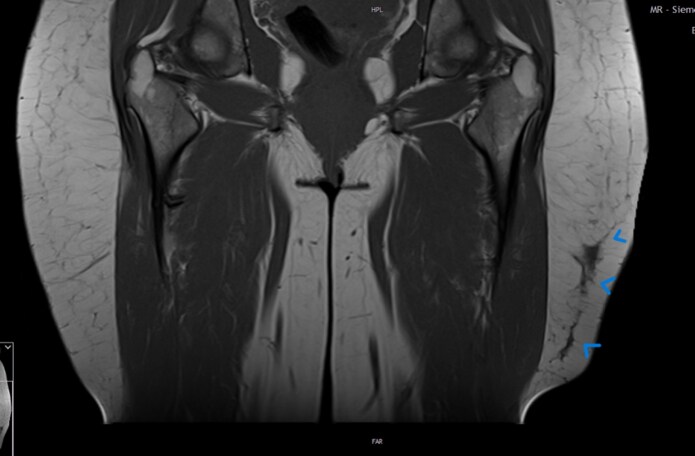
MRI image of the thighs performed at the time of initial presentation, ∼2 years postinjury. Arrows mark the subcutaneous cleft that represents the Morel-Lavallée lesion.

An MRI of the left thigh was conducted at the time of presentation. Imaging revealed a narrow alteration in the subcutaneous tissue, measuring ∼2 to 3 mm in thickness and extending craniocaudally and anteroposteriorly for ∼9 to 10 cm. This finding was consistent with a persistent subcutaneous cleft, suggestive of a chronic MLL or shear injury. Importantly, no space-occupying hematoma or seroma was identified (Figure 3).

In our case, no skin necrosis was present, and an open surgical revision was deemed unnecessary. After weighing the available treatment options, we opted for a deep lipoaspiration of the left thigh extending from the subcutaneous fatty tissue to the fascia level. To minimize the risk of creating large dead spaces and to promote even tissue retraction, liposuction was performed using a multilayer, multichannel technique. This approach allows for the creation of multiple small, intersecting tunnels rather than a single confluent cavity, thereby reducing the likelihood of seroma formation and promoting safer postoperative outcomes.

Two and a half years after the initial trauma, the lipoaspiration procedure was performed on the medial and lateral left thigh in January 2023. A WAL was performed using a tumescent solution supplemented with adrenaline. A total of 3180 mL was infiltrated, and 2050 mL was aspirated from the left leg, a medium-sized cannula (3.8/4.2 mm diameter) was utilized, depending on the device. The tumescent solution contained 0.5 mL of adrenaline (1:1000) per 3 L. No local anesthetic (eg, lidocaine) was added.

**Figure 4.**
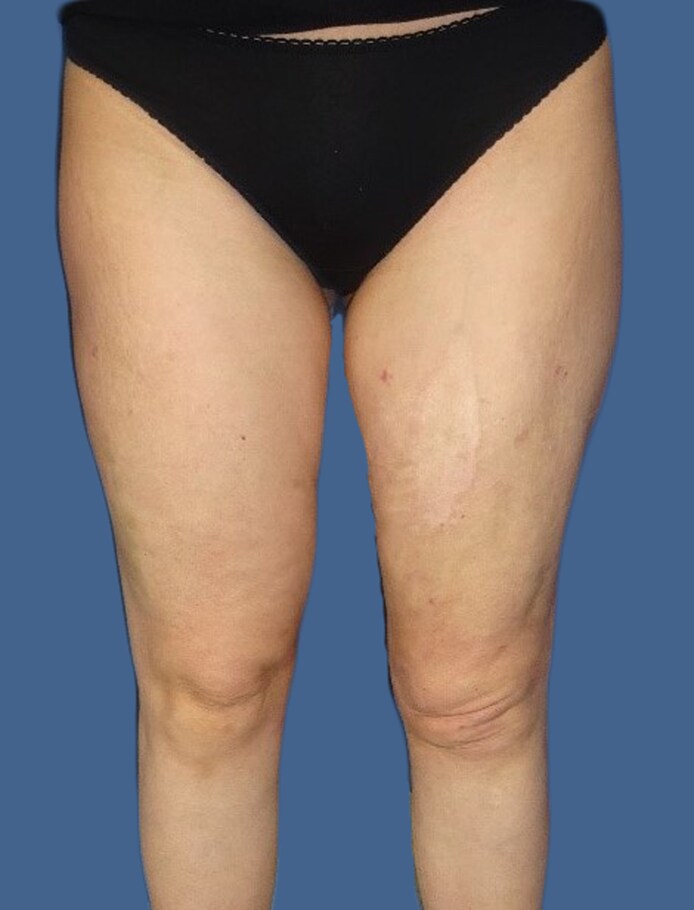
Clinical photograph of a 42-year-old female 3 months after water jet–assisted liposuction to the left thigh with 2050 mL of lipoaspirate. The notable reduction in the previously experienced shifting of the skin and subcutaneous tissue.

The postoperative course was uncomplicated. Subsequent to each lipoaspiration session, the surgical team recommended a minimum 6-week regimen of consistent compression therapy, including the use of flat-knit compression underwear. The patient, however, voluntarily chose to extend compression therapy for a total duration of 3 months, and reported marked functional improvement with a notable reduction in the previously experienced shifting of the skin and subcutaneous tissue (Figure 4).

**Figure 5.**
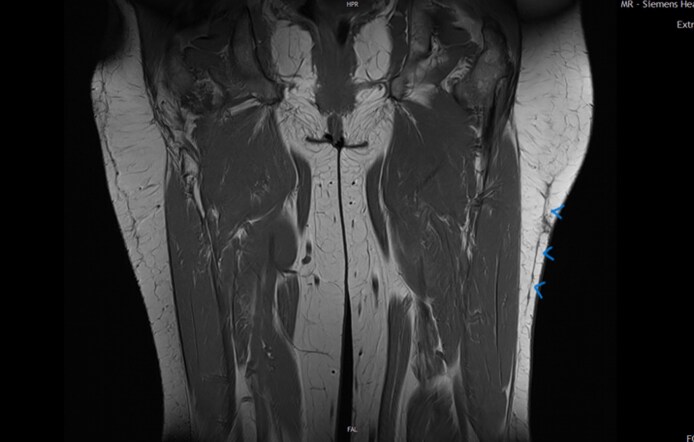
Follow-up MRI conducted 1 year after water jet–assisted liposuction of the left thigh. Arrows mark the subcutaneous cleft that remains visible in the MRI image. The reduction in subcutaneous fatty tissue volume, a linear hypointense signal suggestive of scar formation and no evidence of cavity formation.

At the 1-year follow-up, MRI imaging of the left thigh demonstrated a reduction in subcutaneous fatty tissue volume, particularly in the mid and distal portions of the thigh. Additionally, a linear hypointense signal suggestive of scar formation was noted, and no evidence of cavity formation was observed (Figure 5).

Because of persistent asymmetry between the left and right thighs, and persistent minor displacement of the tissue in relation to the muscle fascia because of an incomplete scarring of the subcutaneous tissue, a second WAL was performed on the left thigh at the patient's request, 3 years after the initial trauma in June 2024. A total of 750 mL of tumescent solution was infiltrated, and ∼750 mL of fat–fluid mixture was aspirated using a blunt and thin to medium-sized liposuction cannula (3.5/3.8 mm diameter), applying a multilayered and multichanneled approach.

**Figure 6.**
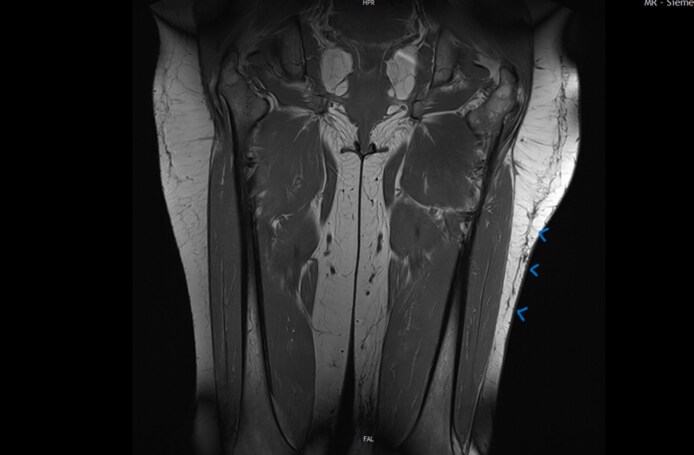
Follow-up MRI conducted 3 months after the second water jet–assisted liposuction of the left thigh. Arrows mark the marginal subcutaneous cleft that remains visible in the MRI image.

**Figure 7.**
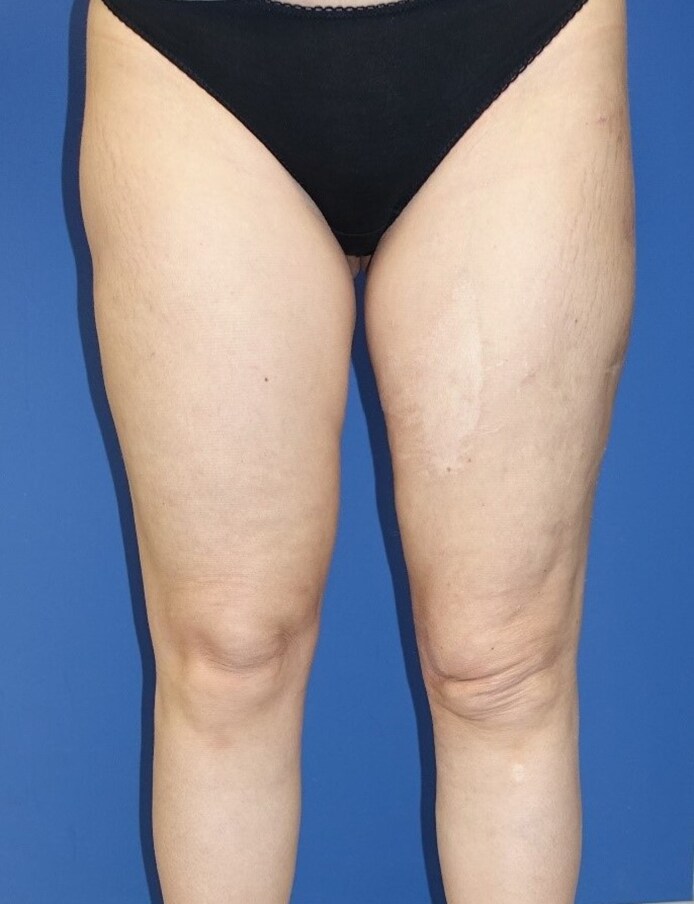
Clinical photograph of a 42-year-old female 3 weeks after secondary water jet–assisted liposuction to the left thigh with 750 mL of lipoaspirate. The improved asymmetry of the thighs. Additionally, the patient reported significant symptomatic improvement and no longer experienced functional limitations.

This second postoperative course was also uneventful. Follow-up MRI showed consistent scar formation within the subcutaneous tissue and only a marginally discernible cavity (Figure 6). The second MRI was performed 3 months after the second surgery. For 6 weeks following surgery, the patient adhered strictly to wearing compression garments. Regular follow-up examinations were conducted until 3 months postoperatively. The patient reported significant symptomatic improvement and no longer experienced functional limitations (Figure 7).

## DISCUSSION

MLLs represent a relatively rare complication of degloving injuries, most frequently occurring over the greater trochanter. They are caused by excessive shear forces, typically resulting from rollover trauma.^1^ However, MLLs have also been described as complications following liposuction procedures involving the thighs. It has been hypothesized that trauma exerted on the subcutaneous tissue by the cannula during liposuction disrupts connective tissue planes, blood vessels, and lymphatics, thereby creating a dead space—particularly when postoperative compression is not employed.^3^ This phenomenon has also been observed in association with dead spaces following lipoabdominoplasties and flap harvesting procedures.^4-7^ Moreover, MLL has been identified as an independent risk factor for the development of surgical site infections in the context of pelvic and acetabular surgery.^8^

When performed, MRI typically demonstrates a well-defined, fusiform fluid collection with signal characteristics that vary depending on the lesion's composition and chronicity. Additional findings may include a surrounding pseudocapsule and a hypointense hemosiderin ring, both of which are commonly associated with chronic MLL.^4^ Internal and peripheral enhancement may be observed, which is consistent with angiogenesis occurring in the lesion wall and septae.^9^ Imaging protocols should include multiplanar T_1_-weighted or proton density sequences, along with fat-saturated T_2_-weighted sequences.^10^

In their study, Kothe et al advocate for early—and, if necessary, repeated—surgical revisions, emphasizing the importance of prompt and adequate evacuation of the soft tissue hematoma resulting from the initial trauma. Debridement is recommended both initially and during planned second-look procedures.^11^ In clinical practice, the cavity between the subcutaneous tissue and the underlying muscle fascia is typically debrided using scrub brushes or similar instruments. Other authors have proposed minimally invasive approaches, such as percutaneous catheter placement application of negative pressure wound therapy, ultrasound-guided aspiration, sclerotherapy, and fibrin sealant injection as effective methods for managing acute MLLs.^12-14^ Additionally, surgical excision of affected soft tissue followed by skin grafting has been described as a potential treatment strategy.^15^

However, in the absence of skin necrosis, preserving native soft tissue is desirable. Persistent soft tissue mobility—especially in areas subject to dynamic stress like the thighs or greater trochanter—can cause functional impairment in addition to aesthetic concerns. In our case, the patient primarily complained of mechanical instability in the thigh that interfered with walking and sitting. The associated asymmetry and shifting tissue further highlighted the functional and cosmetic burden of chronic MLL.

Given these considerations, we opted for a multilayer, multichannel water jet–assisted liposuction (WAL) approach, targeting the fascial plane. This technique was not aimed at simple contour correction, but rather at inducing controlled fibrosis across multiple tissue planes. Our goal was to eliminate the dead space by promoting adherence between tissue layers, while also improving symmetry.

We hypothesize that alternative liposuction modalities, such as power-assisted or ultrasound-assisted liposuction might offer similar benefits. In our view, the decisive factor is not the energy source, but the multiplanar, multidirectional execution of the procedure. This technique enables uniform mechanical disruption and promotes scar formation that stabilizes mobile tissue layers. In our clinical setting, WAL was utilized because of surgeon familiarity and institutional experience. Further comparative studies would be necessary to evaluate the efficacy of other methods.

Although the lesion in our patient was primarily located in the subcutaneous adipose tissue, we believe this technique could be applicable to lesions overlying the fascia, provided that scar induction across the lesion plane is possible. However, further investigation is warranted to validate this hypothesis.

From our perspective, WAL enables targeted and uniform scar induction because of its gentle and controlled tissue dissection. This, in turn, promotes layer adherence and helps eliminate chronic dead space. Whether other multiplanar liposuction techniques offer equivalent outcomes remains to be explored in future research.

Clinically and radiologically, the patient demonstrated significant improvement following the intervention. Although minor surface irregularities remain a known risk in liposuction procedures, the overall result was functionally and aesthetically favorable. MRI follow-up confirmed complete resolution of the lesion and stable integration of the tissue layers.

These findings support the potential role of WAL as a minimally invasive strategy to address chronic MLLs without skin necrosis—achieving both biomechanical stabilization and cosmetic restoration. This case highlights the therapeutic value of scar modulation in treating complex posttraumatic soft tissue conditions.

## CONCLUSIONS

This case demonstrates that WAL can be effectively utilized as a minimally invasive treatment for long-standing MLLs, particularly in cases with pronounced abnormal mobility of the subcutaneous tissue. Through the use of a multilayer, multichannel technique, the procedure promotes scar formation and stabilizes the tissue by eliminating pathological dead space and enhancing adhesion between the subcutaneous fat and underlying fascia. Postoperative MRI confirmed complete obliteration of the fluid-filled cavity, and the patient reported substantial improvements in mobility as well as a more symmetrical and aesthetically satisfying contour. These results highlight the therapeutic potential of scar induction through lipoaspiration, especially when conservative measures have failed.
